# Tendência temporal de detecção, internação hospitalar e mortalidade da sífilis adquirida: análise de série temporal, Brasil, 2011-2023

**DOI:** 10.1590/S2237-96222026v35e20251388.pt

**Published:** 2026-11-02

**Authors:** Sarah Maria Osório de Carvalho, Jesusmar Ximenes Andrade, Fernando Ferraz Nascimento, Malvina Thaís Pacheco Rodrigues, Márcio Dênis Medeiros Mascarenhas

**Affiliations:** 1 Universidade Federal do Piauí, Programa de Pós-graduação em Saúde e Comunidade, Teresina, PI, Brasil

**Keywords:** Sífilis, Diagnóstico, Mortalidade, Morbidade, Estudos de séries temporais, Sífilis, Diagnóstico, Mortalidad, Morbilidad, Estudios de Series Temporales

## Abstract

**Objetivo:**

Analisar a tendência temporal de detecção, internação hospitalar e mortalidade da sífilis adquirida no Brasil no período de 2011 a 2023.

**Métodos:**

Análise de série temporal, com dados secundários do Sistema de Informação de Agravos de Notificação, do Sistema de Informações Hospitalares do Sistema Único de Saúde e do Sistema de Informações sobre Mortalidade. Foram calculadas as taxas de detecção, internação hospitalar e mortalidade segundo região geográfica. Foi utilizado o modelo de regressão de Prais-Winsten para obter a variação percentual anual (VPA) e seus intervalos de confiança de 95% (IC95%) para cada indicador.

**Resultados:**

Foram notificados 1.502.384 casos novos (taxa média de detecção: 56,4 por 100 mil habitantes), 13.017 internações hospitalares (taxa média de internação hospitalar: 0,5 por 100 mil habitantes) e 878 óbitos (taxa média de mortalidade: 0,03 por 100 mil habitantes) por sífilis adquirida no Brasil. A taxa de detecção no Brasil foi crescente (VPA 22,6; IC95% 14,8; 30,9), com maiores incrementos nas regiões Norte (VPA 32,8; IC95% 23,6; 42,7) e Sul (VPA 31,8; IC95% 14,3; 51,3). Observou-se tendência crescente na internação hospitalar (VPA 11,5; IC95% 8,8; 14,3), com destaque para a região Centro-Oeste (VPA 28,3; IC95% 25,6; 32,5). A taxa de mortalidade foi crescente (VPA 0,8; IC95% 0,5; 1,0), com maior incremento na região Sul (VPA 1,1; IC95% 0,6; 1,7).

**Conclusões:**

Observou-se tendência crescente nas taxas de detecção, internação hospitalar e mortalidade da sífilis adquirida no Brasil durante o período estudado. Os achados reforçam a necessidade de respostas locais e intersetoriais fortalecidas.

## Resume

**Objetivo:**

Analizar las tendencias temporales de la detección, la hospitalización y la mortalidad por sífilis adquirida en Brasil entre 2011 y 2023.

**Métodos:**

Análisis de series temporales con datos secundarios del Sistema de Información de Enfermedades de Declaración Obligatoria (*Sistema de Informação de Agravos de Notificação*, SINAN), del Sistema de Información Hospitalaria del Sistema Único de Salud (*Sistema de Informações Hospitalares do Sistema Único de Saúde*, SIH/SUS) y del Sistema de Información sobre Mortalidad (*Sistema de Informações sobre Mortalidade*, SIM). Se calcularon las tasas de detección, hospitalización y mortalidad por región geográfica. Se utilizó el modelo de regresión de Prais-Winsten para estimar la variación porcentual anual (VPA) y sus respectivos intervalos de confianza del 95% (IC95%) para cada indicador.

**Resultados:**

Se notificaron 1.502.384 casos nuevos (tasa media de detección: 56,4 por 100.000 habitantes), 13.017 hospitalizaciones (tasa media de hospitalización: 0,5 por 100.000 habitantes) y 878 defunciones (tasa media de mortalidad: 0,03 por 100.000 habitantes) por sífilis adquirida en Brasil. La tasa de detección mostró una tendencia creciente (VPA 22,6; IC95% 14,8; 30,9), con los mayores incrementos en las regiones Norte (VPA 32,8; IC95% 23,6; 42,7) y Sur (VPA 31,8; IC95%: 14,3; 51,3). También se observó una tendencia creciente en la tasa de hospitalización (VPA 11,5; IC95%: 8,8; 14,3), con destaque para la región Centro-Oeste (VPA 28,3; IC95% 25,6; 32,5). La tasa de mortalidad presentó una tendencia creciente (VPA 0,8; IC95% 0,5; 1,0), con el mayor incremento en la región Sur (VPA 1,1; IC95% 0,6; 1,7).

**Conclusiones:**

Se observaron tendencias crecientes en las tasas de detección, hospitalización y mortalidad por sífilis adquirida en Brasil durante el período estudiado. Los hallazgos refuerzan la necesidad de fortalecer las respuestas locales e intersectoriales.

## Introdução

A sífilis é uma condição prevenível, tratável e curável, mas permanece um desafio relevante para a saúde pública, com crescimento expressivo no número de casos nas últimas décadas, tanto em âmbito nacional quanto internacional. No Brasil, a epidemia de sífilis tem avançado de forma desigual entre as regiões [1]. De 2011 a junho de 2024, foram registrados 1.538.525 casos de sífilis adquirida em pessoas a partir de um ano de idade, sendo a maior concentração de casos na região Sudeste (50%), seguida pelas regiões Sul (22%), Nordeste (14%), Centro-Oeste (7%) e Norte (6%). Destaca-se o crescimento mais acentuado em 2022 e 2023, especialmente no Centro-Oeste (26%) e no Nordeste (20%), o que evidencia a necessidade de estratégias mais efetivas de enfrentamento [2,3]. 

Em resposta a esse panorama, as autoridades de saúde têm implementado diversas medidas de prevenção e controle, como ampliação do acesso ao diagnóstico, oferta de testes rápidos e ações educativas voltadas à população. No entanto, apesar dos esforços empreendidos, a carga da doença permanece elevada, o que reforça a importância do monitoramento contínuo dos indicadores epidemiológicos [4].

A maior parte dos estudos sobre sífilis no Brasil abordam detecção, fatores associados e falhas nos serviços de vigilância e assistência, com destaque para desigualdades regionais. Ainda são escassas, porém, análises voltadas à tendência temporal da doença no país. No cenário brasileiro, observa-se escassez de estudos que explorem de forma integrada registros de casos novos, internações e óbitos relacionados à sífilis adquirida, o que dificulta a formulação de estratégias baseadas em evidências científicas. A análise da evolução temporal da doença permite mensurar a magnitude do problema ao longo dos anos e revelar possíveis lacunas nas políticas de prevenção, diagnóstico e tratamento. Compreender as tendências temporais desses indicadores é fundamental para subsidiar ações de vigilância epidemiológica mais eficazes e fortalecer o enfrentamento da doença [5,6]. 

O estudo teve por objetivo analisar a tendência temporal de detecção, internação hospitalar e mortalidade da sífilis adquirida no Brasil no período de 2011 a 2023.

## Métodos

### Delineamento do estudo

Trata-se de análise de série temporal com dados sobre casos novos, internações hospitalares e óbitos de sífilis adquirida no Brasil e suas regiões no período de janeiro de 2011 a dezembro de 2023.

### Contexto

O estudo foi conduzido com dados de abrangência nacional, em que as unidades de análise foram as cinco regiões geográficas do Brasil (Norte, Nordeste, Sudeste, Sul e Centro-Oeste). Para o cálculo das taxas de detecção, internação e mortalidade, foram utilizadas as estimativas anuais da população residente nessas regiões disponibilizadas pelo Instituto Brasileiro de Geografia e Estatística (IBGE), com a consideração do ano e da região geográfica analisada no período de 2011 a 2023. As populações utilizadas no estudo corresponderam às estimativas intercensitárias e pós-censitárias do IBGE disponíveis para cada ano da série histórica [7].

### Participantes

Foram incluídas no estudo pessoas a partir de um ano de idade, de ambos os sexos, cujos registros constavam como casos novos de sífilis, como internações hospitalares devido à doença em estabelecimentos próprios ou conveniados ao Sistema Único de Saúde (SUS) ou que possuíam menção à sífilis como causa básica de morte.

### Variáveis

As variáveis de interesse foram: sexo (masculino; feminino), faixa etária (1-19; 20-59; ≥60 anos), região geográfica de residência (Norte; Nordeste; Sudeste; Sul; Centro-Oeste) e ano da notificação, da internação ou do óbito (2011-2023). 

### Fontes de dados e mensuração

Utilizaram-se dados secundários anônimos de acesso público. Os casos notificados de sífilis adquirida foram obtidos do Sistema de Informação de Agravos de Notificação (SINAN), as internações hospitalares por sífilis adquirida foram obtidas do Sistema de Informações Hospitalares do Sistema Único de Saúde (SIH/SUS) e os óbitos cuja causa básica tenha sido sífilis adquirida foram obtidos do Sistema de Informações sobre Mortalidade (SIM). As estimativas populacionais empregadas como denominador para cálculo das taxas foram obtidas a partir do IBGE. Todos os dados utilizados no estudo foram obtidos por meio do tabulador TabNet através do portal eletrônico do Departamento de Informação e Informática do Sistema Único de Saúde (DATASUS).

Para identificação das internações hospitalares e dos óbitos por sífilis adquirida foram utilizados os códigos A51, A52 e A53 da Classificação Internacional de Doenças - 10ª revisão (CID-10). No SIH/SUS, foram selecionadas as internações hospitalares cujo diagnóstico principal correspondeu a esses códigos e, no SIM, os óbitos cuja causa básica foi classificada da mesma forma.

A taxa de detecção foi obtida a partir da razão entre o número de casos novos diagnosticados de sífilis adquirida, por local e por ano, e a população residente estimada para a respectiva região geográfica e o ano de ocorrência, segundo dados do (IBGE), multiplicada por 100 mil habitantes. A taxa de internação hospitalar foi calculada a partir da divisão do número de internações hospitalares com diagnóstico de sífilis adquirida pela população residente estimada por região geográfica e por ano de ocorrência, segundo dados do (IBGE), multiplicada por 100 mil habitantes. Para a taxa de mortalidade, considerou-se a razão entre o número de óbitos cuja causa básica foi atribuída à sífilis adquirida e a população residente no mesmo recorte espacial e temporal multiplicada por 100 mil habitantes. 

### Viés

Por utilizar dados secundários provenientes do SINAN, do SIH/SUS e do SIM, o estudo esteve sujeito a possíveis vieses relacionados a subnotificação, incompletude e inconsistências dos registros. Mudanças na vigilância epidemiológica, na ampliação do acesso ao diagnóstico e nas variações na cobertura dos sistemas ao longo do período também podem ter influenciado as tendências observadas. Para minimizar tais limitações, foram utilizados dados oficiais disponibilizados pelo DATASUS, com padronização das variáveis, dos indicadores e dos procedimentos de análise.

### Tamanho do estudo

Foram analisados dados agregados por regiões geográficas e no Brasil como um todo referentes aos registros de casos novos, internações hospitalares e óbitos por sífilis adquirida no período de 2011 a 2023. Respectivamente, os dados foram obtidos do SINAN, do SIH/SUS e do SIM. As taxas de detecção, internação hospitalar e mortalidade foram calculadas com a utilização das estimativas populacionais anuais do IBGE como denominador multiplicadas por 100 mil habitantes.

### Métodos estatísticos

As taxas médias de detecção, internação hospitalar e mortalidade por sífilis adquirida foram obtidas por meio do somatório das taxas anuais observadas em cada variável no período de 2011 a 2023, com a divisão do resultado pelo número total de anos analisados (n=13). Obteve-se, então, a média aritmética simples das taxas para cada variável, o que permitiu visão geral da magnitude média dos indicadores no período estudado. Devido à baixa frequência de óbitos por sífilis adquirida, a taxa de mortalidade não foi desagregada por sexo e por faixa etária para cada região geográfica. 

Para análise da tendência temporal das taxas de detecção, internação hospitalar e mortalidade por sífilis adquirida, utilizou-se o modelo de regressão linear generalizada de Prais-Winsten, método adequado para séries temporais com autocorrelação de primeira ordem. O período do estudo (2011-2023) foi considerado como variável independente (X), enquanto o logaritmo de base 10 das taxas de detecção, internação hospitalar e mortalidade por sífilis adquirida, geral e desagregadas, foi a variável dependente (Y). Calculou-se a variação percentual anual (VPA) com seus respectivos intervalos de confiança de 95% (IC95%) de cada indicador analisado. A VPA foi calculada a partir do coeficiente β obtido pela regressão de Prais-Winsten aplicada às séries temporais log-transformadas (log_10_ das taxas) [8]. Para isso, utilizou-se a fórmula: VPA=(-1+10^β^)×100. Essa fórmula expressa a mudança percentual média anual de cada indicador ao longo da série temporal analisada. O valor de β representa o coeficiente da regressão. Os valores dos IC95% da VPA foram calculados da mesma forma, com aplicação da fórmula aos limites inferior e superior do coeficiente de regressão (β). 

O p-valor, obtido para o coeficiente de regressão (β), foi utilizado para determinar a significância estatística da tendência observada, com adoção do nível de significância quando o p-valor<0,050. As tendências foram classificadas como crescente, decrescente ou estacionária, conforme o sinal e a significância estatística do coeficiente de regressão (β). Considerou-se: tendência crescente quando o coeficiente de regressão (β) apresentou valor positivo e p-valor<0,050 (ou IC95% totalmente acima de zero); tendência decrescente quando o coeficiente de regressão (β) foi negativo e p-valor<0,050 (ou IC95% totalmente abaixo de zero); e tendência estacionária quando o p-valor≥0,050 (ou o IC95% incluiu o zero), o que indicava ausência de variação estatisticamente significativa ao longo do período analisado.

Foi utilizado o programa Microsoft Excel 2019 para Windows na etapa de organização do banco de dados e do cálculo das taxas de detecção, internação hospitalar e mortalidade. As demais análises estatísticas foram realizadas no software Stata, versão 16.0.

## Resultados

No Brasil, no período de janeiro de 2011 a dezembro de 2023, foram notificados 1.502.384 casos novos de sífilis adquirida (taxa média de detecção: 56,4 por 100 mil habitantes), 13.017 internações hospitalares de pacientes acometidos (taxa média de internação: 0,5 por 100 mil habitantes) e 878 óbitos (taxa média de mortalidade: 0,03 por 100 mil habitantes) em decorrência da doença.

A taxa de detecção no Brasil apresentou tendência crescente (VPA 22,6; IC95% 14,8; 30,9). O crescimento foi igualmente crescente entre mulheres (VPA 22,1; IC95% 13,7;31,1) e homens (VPA 22,6; IC95% 15,1; 30,6). Esse aumento foi consistente entre todas as faixas etárias avaliadas, com maior crescimento entre pessoas de 1 a 19 anos (VPA 26,6; IC95% 14,0; 40,6) (Tabela 1).

A taxa de detecção também apresentou tendência de crescimento segundo as regiões brasileiras. As maiores taxas de aumento foram verificadas na região Norte (VPA 32,8; IC95% 23,6; 42,7), seguida pela região Sul (VPA 31,8; IC95% 14,3; 51,3). As análises estratificadas por sexo e por faixa etária em cada região também evidenciaram tendências crescentes na detecção de sífilis adquirida, com destaque para a região Sul, onde os aumentos foram crescentes, com incremento médio em torno de 30,6% a 32,4% em todos os estratos analisados (Tabela 1).

A taxa de internação hospitalar por sífilis adquirida no Brasil apresentou tendência crescente no país como um todo (VPA 11,5; IC95% 8,8; 14,3), especialmente entre homens (VPA 12,0; IC95% 9,9; 14,2) e pessoas com 60 anos ou mais (VPA 13,9; IC95% 11,7; 16,3) (Tabela 2). 

Houve tendência de crescimento da taxa de internação hospitalar em todas as regiões brasileiras. O maior aumento proporcional anual foi registrado no Centro-Oeste (VPA 28,3; IC95% 25,6; 32,5), seguido pela região Nordeste (VPA 12,4; IC95% 7,7; 17,3). Em algumas subpopulações, no entanto, foram observadas tendências estacionárias. Na região Norte, a taxa de internação hospitalar manteve-se estacionária na população de 60 anos de idade ou mais (VPA 12,5; IC95% -0,8; 27,6). Tendência de estabilidade também foi observada entre pessoas de 1 a 19 anos de idade (VPA 6,4; IC95% -2,4; 16,1) na região Nordeste e entre pessoas de 1 a 19 anos de idade (VPA 1,1; IC95% -5,0; 7,5) na região Sul. Nas demais regiões e subgrupos, observou-se tendência crescente na taxa de internação hospitalar. Na região Centro-Oeste, o maior incremento foi observado no sexo feminino (VPA 35,7; IC95% 29,4; 42,2) e entre pessoas de 20 a 59 anos de idade (VPA 28,7; IC95% 25,0; 32,5) (Tabela 2).

**Tabela 1:** Variação percentual anual (VPA), intervalos de confiança de 95% (IC95%) e tendência temporal da taxa de detecção de sífilis adquirida, por 100 mil habitantes, segundo sexo, faixa etária e região. Brasil, 2011-2023 (n=1.502.384)^a^

Variáveis	Total (n)	Taxa média	VPA (IC95%)	p-valor	Tendência
Brasil	1.502.384	56,4	22,6 (14,8; 30,9)	<0,001	Crescente
Sexo					
Feminino	587.660	43,1	22,1 (13,7; 31,1)	<0,001	Crescente
Masculino	909.368	69,9	22,6 (15,1; 30,6)	<0,001	Crescente
Faixa etária (anos)					
1-19	147.088	19,7	26,6 (14,0; 40,6)	<0,001	Crescente
20-59	1.224.454	79,5	22,3 (14,8; 30,2)	<0,001	Crescente
≥60	125.486	33,7	16,2 (10,0; 22,7)	<0,001	Crescente
Norte	94.430	40,9	32,8 (23,6; 42,7)	<0,001	Crescente
Sexo					
Feminino	37.892	33,0	31,3 (22,6; 40,7)	<0,001	Crescente
Masculino	56.291	48,5	33,5 (23,5; 44,1)	<0,001	Crescente
Faixa etária (anos)					
1-19	10.627	13,3	32,1 (22,6; 42,4)	<0,001	Crescente
20-59	76.805	59,6	31,7 (22,7; 41,5)	<0,001	Crescente
≥60	6.802	31,0	34,5 (23,3; 46,7)	<0,001	Crescente
Nordeste	215.944	29,9	27,9 (18,5; 38,1)	<0,001	Crescente
Sexo					
Feminino	87.913	23,7	24,1 (14,4; 34,6)	<0,001	Crescente
Masculino	127.080	36,2	30,8 (20,7; 41,8)	<0,001	Crescente
Faixa etária (anos)					
1-19	20.922	9,7	27,4 (14,9; 41,2)	<0,001	Crescente
20-59	175.505	42,6	26,9 (17,9; 36,5)	<0,001	Crescente
≥60	18.843	19,6	33,2 (21,6; 45,9)	<0,001	Crescente
Sudeste	754.138	67,4	18,0 (12,4; 23,9)	<0,001	Crescente
Sexo					
Feminino	280.572	48,7	17,7 (11,8; 23,9)	<0,001	Crescente
Masculino	470.989	86,9	17,9 (12,4; 23,7)	<0,001	Crescente
Faixa etária (anos)					
1-19	73.851	25,4	23,9 (13,1; 35,8)	<0,001	Crescente
20-59	612.441	93,7	18,1 (12,8; 23,6)	<0,001	Crescente
≥60	65.269	38,5	9,5 (4,4; 14,9)	<0,001	Crescente
Sul	328.964	85,3	31,8 (14,3; 51,3)	0,001	Crescente
Sexo					
Feminino	143.424	72,8	31,7 (14,0; 52,3)	0,002	Crescente
Masculino	184.656	97,7	31,0 (14,1; 50,4)	0,001	Crescente
Faixa etária (anos)					
1-19	31.365	31,5	32,4 (12,0; 56,4)	0,004	Crescente
20-59	269.247	119,8	31,2 (14,5; 50,3)	0,001	Crescente
≥60	27.468	44,6	30,6 (13,3; 50,6)	0,002	Crescente
Centro-Oeste	108.908	52,2	28,5 (19,0; 38,9)	<0,001	Crescente
Sexo					
Feminino	37.859	35,9	26,5 (17,4; 36,4)	<0,001	Crescente
Masculino	70.352	68,2	29,4 (19,2; 40,5)	<0,001	Crescente
Faixa etária					
1-19	10.353	17,2	26,9 (16,1; 38,7)	<0,001	Crescente
20-59	90.721	73,5	28,7 (18,89; 39,4)	<0,001	Crescente
≥60	7.137	28,3	23,6 (17,0; 30,5)	<0,001	Crescente

a A diferença entre o total de registros e a soma das categorias de sexo e de faixa etária decorre da existência de registros com informação ignorada ou não preenchida.

**Tabela 2:** Variação percentual anual (VPA), intervalos de confiança de 95% (IC95%) e tendência temporal da taxa de internação hospitalar por sífilis adquirida, por 100 mil habitantes, segundo sexo, faixa etária e região. Brasil, 2011-2023 (n=13.036)^a^

Variáveis	Total (n)	Taxa média	VPA (IC95%)	p-valor	Tendência
Brasil	13.017	0,5	11,5 (8,8; 14,3)	<0,001	Crescente
Sexo					
Feminino	6.280	0,5	10,9 (7,5; 14,4)	<0,001	Crescente
Masculino	6.737	0,5	12,0 (9,9; 14,2)	<0,001	Crescente
Faixa etária (anos)					
1-19	1.427	0,2	8,5 (4,0; 13,2)	0,001	Crescente
20-59	9.264	0,6	11,1 (8,2; 14,1)	<0,001	Crescente
≥60	2.326	0,6	13,9 (11,7; 16,3)	<0,001	Crescente
Norte	534	0,2	11,0 (4,9; 17,4)	0,002	Crescente
Sexo					
Feminino	280	0,3	9,9 (1,3; 19,3)	0,026	Crescente
Masculino	254	0,2	12,1 (8,1; 16,3)	<0,001	Crescente
Faixa etária (anos)					
1-19	108	0,1	10,1 (2,2; 18,6)	0,016	Crescente
20-59	367	0,3	10,6 (5,7; 15,8)	0,001	Crescente
≥60	59	0,3	12,5 (-0,8; 27,6)	0,063	Estacionária
Nordeste	2.477	0,4	12,4 (7,68; 17,3)	<0,001	Crescente
Sexo					
Feminino	1.465	0,4	9,3 (3,5; 15,4)	0,004	Crescente
Masculino	1.012	0,3	17,6 (14,1; 21,4)	<0,001	Crescente
Faixa etária (anos)					
1-19	373	0,2	6,4 (-2,4; 16,1)	0,142	Estacionária
20-59	1.740	0,5	12,2 (7,4; 17,2)	<0,001	Crescente
≥60	364	0,4	20,7 (13,0; 28,9)	<0,001	Crescente
Sudeste	6.488	0,6	9,2 (6,2; 12,3)	<0,001	
Sexo					
Feminino	2.753	0,5	8,4 (4,0; 12,9)	0,001	Crescente
Masculino	3.735	0,7	9,9 (7,3; 12,5)	<0,001	Crescente
Faixa etária (anos)					
1-19	571	0,2	6,9 (1,2; 13,0)	0,022	Crescente
20-59	4.659	0,7	8,6 (5,4; 12,0)	<0,001	Crescente
≥60	1.258	0,7	11,0 (8,9; 13,2)	<0,001	Crescente
Sul	2.169	0,6	12,3 (7,4; 17,3)	<0,001	
Sexo					
Feminino	921	0,5	11,2 (5,2; 17,4)	0,001	Crescente
Masculino	1.248	0,7	12,6 (9,0; 16,3)	<0,001	Crescente
Faixa etária (anos)					
1-19	187	0,2	1,1 (-5,0; 7,5)	0,707	Estacionária
20-59	1.483	0,7	11,0 (6,4; 15,7)	<0,001	Crescente
≥60	499	0,8	19,3 (12,9; 26,1)	<0,001	Crescente
Centro-Oeste	1.349	0,6	29,0 (25,6; 32,5)	<0,001	Crescente
Sexo					
Feminino	861	0,8	35,7 (29,4; 42,2)	<0,001	Crescente
Masculino	488	0,5	21,9 (18,6; 25,4)	<0,001	Crescente
Faixa etária (anos)					
1-19	189	0,3	39,7 (29,6; 50,6)	<0,001	Crescente
20-59	1.015	0,8	28,7 (25,0; 32,5)	<0,001	Crescente
≥60	146	0,6	17,3 (14,5; 20,2)	<0,001	Crescente

a A diferença entre o total de registros e a soma das categorias de sexo e de faixa etária decorre da existência de registros com informação ignorada ou não preenchida.

A mortalidade por sífilis adquirida apresentou tendência crescente no Brasil (VPA 0,8; IC95% 0,5; 1,0), tanto entre mulheres (VPA 0,4; IC95% 0,3; 0,6) quanto entre homens (VPA 1,2; IC95% 0,8; 1,6). Observou-se aumento tanto entre adultos de 20 a 59 anos (VPA 0,7; IC95% 0,4; 0,9) quanto entre idosos com 60 anos ou mais (VPA 2,0; IC95% 1,0; 3,1). Por outro lado, entre adolescentes e jovens de 1 a 19 anos, a tendência foi estacionária (VPA 0,01; IC95% -0,03; 0,05). Houve aumento na taxa da mortalidade para quatro regiões, sendo o maior incremento observado na região Sul (VPA 1,1; IC95% 0,6; 1,7), seguida do Centro-Oeste (VPA 1,1; IC95% 0,7; 1,6) (Tabela 3).

A Figura 1 apresenta a evolução dos indicadores epidemiológicos relacionados à sífilis adquirida no Brasil. Observa-se tendência crescente na taxa de detecção de sífilis adquirida, com valores mais elevados no sexo masculino e no grupo de pessoas com 20-59 anos de idade (Figura 1-A). A taxa de internação apresentou crescimento ao longo do tempo analisado, principalmente a partir de 2016, com valores mais elevados no sexo masculino e no grupo de pessoas com 60 anos ou mais (Figura 1-B). A mortalidade por sífilis apresentou evolução crescente, com maiores valores no sexo masculino e no grupo de pessoas a partir de 60 anos de idade (Figura 1-C). Os indicadores de detecção e de hospitalização apresentaram tendência decrescente em 2020.

**Tabela 3:** Variação percentual anual (VPA), intervalos de confiança de 95% (IC95%) e tendência temporal da taxa de mortalidade por sífilis adquirida, por 100 mil habitantes, segundo sexo, faixa etária e região. Brasil, 2011-2023 (n=878)^a^

Variáveis	Total (n)	Taxa média	VPA (IC95%)	p-valor	Tendência
Brasil	878	0,03	0,8 (0,5; 1,0)	<0,001	Crescente
Sexo					
Feminino	276	0,02	0,4 (0,3; 0,6)	<0,001	Crescente
Masculino	592	0,05	1,2 (0,8; 1,6)	<0,001	Crescente
Faixa etária (anos)					
1-19	16	0,002	0,01 (-0,03; 0,05)	0,626	Estacionária
20-59	418	0,03	0,7 (0,5; 1,0)	<0,001	Crescente
≥60	434	0,12	2,0 (1,0; 3,1)	0,001	Crescente
Norte	56	0,02	0,7 (0,5; 1,0)	<0,001	Crescente
Nordeste	202	0,03	0,5 (0,3; 0,7)	<0,001	Crescente
Sudeste	392	0,04	0,9 (0,5; 1,2)	<0,001	Crescente
Sul	154	0,04	1,2 (0,6; 1,7)	0,001	Crescente
Centro-Oeste	74	0,04	1,1 (0,8; 1,5)	<0,001	Crescente

a A diferença entre o total de registros e a soma das categorias de sexo e de faixa etária decorre da existência de registros com informação ignorada ou não preenchida.

**Figura 1:**
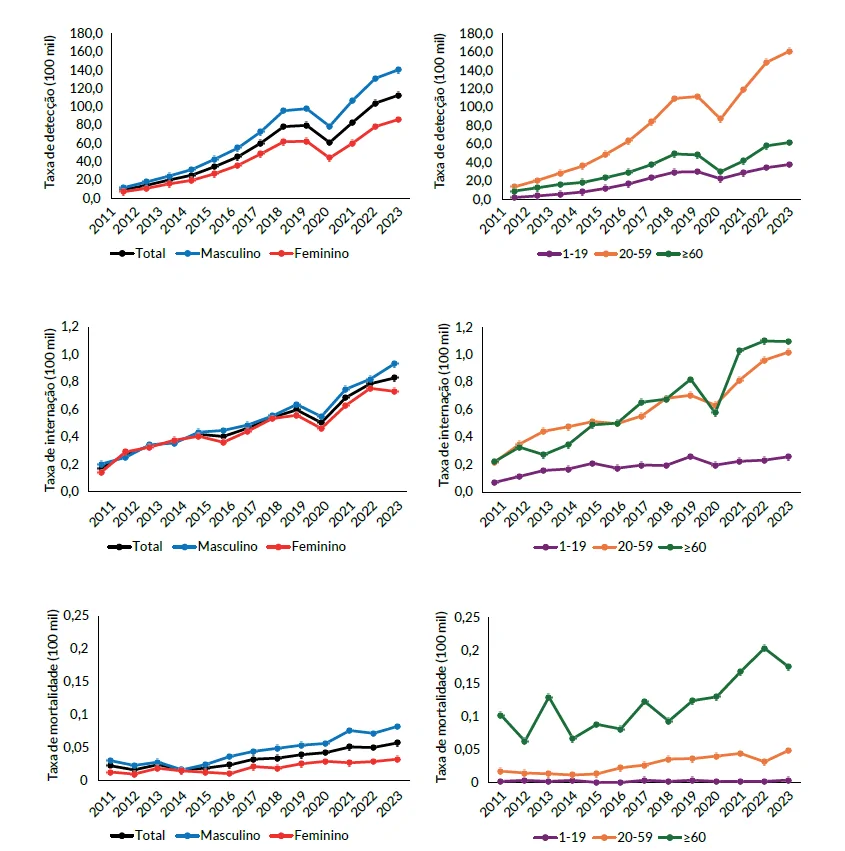
Evolução temporal das taxas de detecção, internação hospitalar e mortalidade por sífilis adquirida no Brasil entre 2011-2023 com: taxa de detecção segundo sexo (A1), taxa de detecção segundo faixa etária (A2), taxa de internação hospitalar segundo sexo (B1), taxa de internação hospitalar segundo faixa etária (B2), taxa de mortalidade segundo sexo (C1) e taxa de mortalidade segundo faixa etária (C2). Brasil, 2011-2023

## Discussão

A análise dos indicadores epidemiológicos da sífilis adquirida no Brasil evidenciou tendência de aumento de detecção, internação hospitalar e mortalidade ao longo do período estudado, 2011-2023, com diferenças na magnitude entre as regiões do país. Os achados demonstraram a persistência da sífilis como importante problema de saúde pública, o que sugere limitações nas estratégias de prevenção, diagnóstico e tratamento da infecção.

Na região Sul, observou-se o maior crescimento proporcional na detecção de sífilis adquirida no país, além de aumento nas taxas de internação e mortalidade. Esses achados indicaram um quadro de alta circulação do *Treponema pallidum*, mesmo em um contexto de ampla cobertura da atenção primária à saúde e maior estruturação dos serviços de saúde. O comportamento observado sugeriu possíveis falhas na implementação das ações de prevenção e no manejo oportuno da infecção, inclusive desafios relacionados ao diagnóstico precoce e à adesão terapêutica, o que evidenciou a necessidade de aprimoramento das estratégias locais de controle. Estudos prévios já destacaram a complexidade da sífilis em regiões desenvolvidas, com atribuição do problema à disponibilidade de serviços de saúde para diagnóstico e tratamento, a comportamentos de risco persistentes e à subnotificação em alguns estratos [9,10]. 

O Sudeste também apresentou crescimento expressivo nos três indicadores, com aumento acentuado de casos notificados e de desfechos mais graves, como internações e óbitos, o que corroborou estudo realizado na mesma região [11]. Apesar de ela contar com maior capacidade instalada para diagnóstico e tratamento, fatores como alta densidade populacional, desigualdades sociais, estigma e descontinuidade no abastecimento de insumos dificultaram o controle da infecção, além de contribuírem para o aumento da transmissibilidade. Alguns estudos [12,13] reforçaram que a fragmentação do cuidado e as barreiras socioeconômicas tem impedido o acesso efetivo à prevenção e ao tratamento mesmo em centros urbanos com infraestrutura, o que leva ao aumento da dinâmica de transmissão. 

No Centro-Oeste, a análise revelou crescimento significativo na detecção, na internação e na mortalidade por sífilis adquirida. Esse cenário pode ter estado associado a intensos processos migratórios, à urbanização acelerada e à insuficiente expansão dos serviços de saúde, principalmente em áreas rurais e periferias urbanas [14]. Estudo recente enfatizou que o crescimento desordenado e a precarização dos serviços de saúde em áreas de fronteira e de expansão urbana frequentemente resultaram em maiores taxas de infecções sexualmente transmissíveis, o que validou os achados desta análise [15]. 

A região Norte também apresentou crescimento expressivo, especialmente na detecção da infecção. O aumento nos registros pode refletir tanto a ampliação do acesso ao diagnóstico quanto o avanço real da circulação da doença. Estudos afirmaram que desafios estruturais como baixa cobertura da atenção primária à saúde, dispersão populacional e dificuldades de acesso aos serviços de saúde em áreas remotas e em comunidades tradicionais tornam a região particularmente vulnerável, o que reforçou a necessidade de estratégias de controle adaptadas às realidades locais [13,16,17]. 

O Nordeste, por sua vez, apresentou crescimento nas taxas de detecção e mortalidade, mas manteve tendência de estabilidade nas internações hospitalares. Embora os números absolutos da região sejam inferiores aos das demais, é necessário cautela na interpretação dos dados, uma vez que subnotificação e acesso limitado aos serviços de saúde podem mascarar a real magnitude da infecção. A desigualdade socioeconômica, as barreiras geográficas e a precariedade dos serviços de vigilância epidemiológica podem ter contribuído para o subdiagnóstico e, consequentemente, para a manutenção de altas taxas de transmissão [18,15,19]. 

A série temporal demonstrou a redução dos indicadores de detecção e internação no ano de 2020, aspecto importante que coincidiu com o primeiro ano da pandemia de covid-19. Essa diminuição pode ter estado relacionada aos impactos da pandemia sobre os serviços de saúde, o que inclui redução da testagem, dificuldades de acesso aos serviços, interrupções no acompanhamento dos casos e possível subnotificação. A retomada do crescimento dos indicadores nos anos subsequentes sugeriu a manutenção da circulação ativa da sífilis no país [20]. 

A elevação das taxas nacionais de detecção, internação e mortalidade indicou a importância do fortalecimento das políticas públicas direcionadas ao enfrentamento da sífilis adquirida. Destaca-se a importância de ampliar o acesso ao diagnóstico precoce, assegurar a disponibilidade contínua de medicamentos como a penicilina benzatina e implementar ações educativas e preventivas específicas para grupos populacionais vulneráveis. Tornou-se relevante, ademais, qualificar as ações de monitoramento e assistência em saúde, com consideração das especificidades territoriais e da articulação entre os diferentes serviços da rede de atenção à saúde [21]. 

A análise dos dados por faixa etária e sexo também revelou tendências preocupantes. Observou-se aumento mais acentuado na detecção de sífilis adquirida entre adolescentes e jovens de 1 a 19 anos, o que evidenciou maior vulnerabilidade dessa população, comumente relacionada ao início precoce da vida sexual, ao uso irregular de preservativos e ao acesso restrito a serviços de saúde sexual e reprodutiva. Os adultos de 20 a 59 anos e os idosos também apresentaram elevação significativa, o que indicou a permanência de comportamentos de risco nessas faixas etárias e a ausência de estratégias preventivas direcionadas ao envelhecimento [22,9].

Os resultados de internações hospitalares também reforçaram a gravidade da situação, com aumento significativo em praticamente todos os grupos etários e sexos, o que sinalizou falhas no diagnóstico oportuno e dificuldades no acesso ao tratamento adequado. A elevação das taxas de mortalidade entre adultos e idosos, particularmente entre homens, destacou desigualdades persistentes nos padrões de cuidado, o que sugeriu maior resistência masculina em buscar atendimento médico e em aderir ao tratamento. Esses achados apontaram para a importância de intervenções específicas para cada grupo populacional, com a consideração das suas particularidades epidemiológicas, sociais e culturais, a fim de conter a progressão da sífilis adquirida no Brasil [23].

Este estudo apresentou limitações inerentes ao uso de dados secundários provenientes de sistemas nacionais de informação em saúde, sujeitos a subnotificação, inconsistências de preenchimento e diferenças regionais na qualidade dos registros. Essas limitações podem ter influenciado a estimativa das taxas de detecção, internação e mortalidade por sífilis adquirida, bem como ter comprometido a comparabilidade entre as macrorregiões brasileiras. Os impactos da pandemia de covid-19 sobre os serviços de saúde, especialmente em 2020, também podem ter contribuído para redução da testagem, atraso diagnóstico e possível subregistro dos casos, o que influenciou a interpretação das tendências temporais observadas.

A análise das taxas de detecção, internação e mortalidade por sífilis adquirida no Brasil entre 2011 e 2023 demonstrou aumento dos indicadores epidemiológicos na maioria das regiões brasileiras, com variações na magnitude observada entre os indicadores e as regiões. Os dados evidenciaram que a sífilis permanece como um grave problema de saúde pública, com impacto significativo em termos de morbidade e mortalidade, especialmente nas regiões Sul, Sudeste e Centro-Oeste, onde se observaram os maiores índices acumulados e as maiores variações percentuais anuais. A inflexão nas notificações em 2020, atribuída à pandemia de covid-19, reforçou a vulnerabilidade dos sistemas de vigilância e de diagnóstico frente a crises sanitárias, o que evidenciou a necessidade de estratégias resilientes e sustentáveis. A elevação significativa das taxas nacionais e regionais apontou para falhas persistentes nas ações de prevenção, testagem e tratamento, além de para insuficiente integração entre políticas públicas e ações intersetoriais.

Os resultados demonstraram tendência de aumento de detecção, internação hospitalar e mortalidade por sífilis adquirida no Brasil entre 2011 e 2023, com diferenças regionais importantes. Os achados reforçaram a relevância da sífilis como problema de saúde pública e destacaram a necessidade de aprimoramento das ações de prevenção, de diagnóstico, de vigilância e de tratamento da infecção no país.

## Data Availability

Os dados estão disponíveis integralmente em https://doi.org/10.48331/SCIELODATA.CWVUG8.
