# Supplementary material for: Tendência temporal de detecção, internação hospitalar e mortalidade da sífilis adquirida: análise de série temporal, Brasil, 2011-2023
Source: Epidemiol Serv Saude. 2026 Nov 2;35:e20251388. [Article in Portuguese] doi: 10.1590/S2237-96222026v35e20251388.pt (PMC13730452; doi:10.1590/S2237-96222026v35e20251388.pt)
Supplement: Translation

# Translation: Temporal trends in acquired syphilis detection, hospitalizations, and mortality: a time series analysis, Brazil, 2011-2023

**DOI:** 10.1590/S2237-96222026v35e20251388.en

**Authors:** Sarah Maria Osório de Carvalho, Jesusmar Ximenes Andrade, Fernando Ferraz Nascimento, Malvina Thaís Pacheco Rodrigues, Márcio Dênis Medeiros Mascarenhas

**Affiliations:** 1 Universidade Federal do Piauí, Programa de Pós-graduação em Saúde e Comunidade, Teresina, PI, Brazil

**Keywords:** Syphilis, Diagnosis, Mortality, Morbidity, Time Series Analysis

## Abstract

**Objective:**

To analyze temporal trends in acquired syphilis detection, hospitalizations, and mortality in Brazil from 2011 to 2023.

**Methods:**

Time series analysis using secondary data from the Notifiable Diseases Information System (*Sistema de Informação de Agravos de Notificação*, SINAN), the National Hospital Information System of the Brazilian Unified Health System (*Sistema de Informações Hospitalares do Sistema Único de Saúde*, SIH/SUS), and the Mortality Information System (*Sistema de Informações sobre Mortalidade*, SIM). Detection, hospitalization, and mortality rates were calculated according to geographic region. Prais-Winsten regression models were used to estimate the annual percent change (APC) and corresponding 95% confidence intervals (95%CI) for each indicator.

**Results:**

A total of 1,502,384 new cases (mean detection rate: 56.4 per 100,000 inhabitants), 13,017 hospitalizations (mean hospitalization rate: 0.5 per 100,000 inhabitants), and 878 deaths (mean mortality rate: 0.03 per 100,000 inhabitants) due to acquired syphilis were reported in Brazil. The detection rate in Brazil showed an increasing trend (APC 22.6; 95%CI 14.8; 30.9), with the largest increases observed in the North (APC 32.8; 95%CI 23.6; 42.7) and South (APC 31.8; 95%CI 14.3; 51.3) regions. An increasing trend was also observed in hospitalization rates (APC 11.5; 95%CI: 8.8; 14.3), particularly in the Central-West region (APC 28.3; 95%CI 25.6; 32.5). The mortality rate showed an increasing trend (APC 0.8; 95%CI 0.5; 1.0), with the greatest increase observed in the South region (APC 1.1; 95%CI 0.6; 1.7).

**Conclusions:**

Increasing trends were observed in acquired syphilis detection, hospitalization, and mortality rates in Brazil throughout the study period. These findings reinforce the need for strengthened local and intersectoral responses.

## Introduction

Syphilis is a preventable, treatable, and curable condition; however, it remains a major public health challenge, with a marked increase in the number of cases over recent decades, both nationally and internationally. In Brazil, the syphilis epidemic has progressed unevenly across regions [1]. From 2011 to June 2024, a total of 1,538,525 cases of acquired syphilis were reported among individuals aged 1 year or older, with the highest concentration of cases in the Southeast region (50%), followed by the South (22%), Northeast (14%), Central-West (7%), and North (6%) regions. A more pronounced increase was observed in 2022 and 2023, particularly in the Central-West (26%) and Northeast (20%) regions, highlighting the need for more effective response strategies [2,3]. 

In response to this scenario, health authorities have implemented several prevention and control measures, including expanded access to diagnosis, provision of rapid tests, and educational initiatives targeting the population. Nevertheless, despite these efforts, the burden of disease remains high, reinforcing the importance of continuous monitoring of epidemiological indicators [4].

Most studies on syphilis in Brazil have focused on detection, associated factors, and shortcomings in surveillance and healthcare services, with particular emphasis on regional inequalities. However, studies addressing the temporal trends of the disease in the country remain scarce. In the Brazilian context, there is a lack of studies that comprehensively examine records of new cases, hospitalizations, and deaths related to acquired syphilis, limiting the development of evidence-informed strategies. Analyzing the temporal evolution of the disease makes it possible to assess the magnitude of the problem over time and identify potential gaps in prevention, diagnosis, and treatment policies. Understanding the temporal trends of these indicators is essential to support more effective epidemiological surveillance actions and strengthen the response to the disease [5,6]. 

The objective of this study was to analyze the temporal trends in acquired syphilis detection, hospitalizations, and mortality in Brazil from 2011 to 2023.

## Methods

### Study design

This was a time series analysis using data on new cases, hospitalizations, and deaths due to acquired syphilis in Brazil and its geographic regions from January 2011 to December 2023.

### Setting

The study was conducted using nationwide data, with the five geographic regions of Brazil (North, Northeast, Southeast, South, and Central-West) as the units of analysis. Annual estimates of the resident population for these regions, provided by the Brazilian Institute of Geography and Statistics (*Instituto Brasileiro de Geografia e Estatística*, IBGE), were used to calculate detection, hospitalization, and mortality rates, according to the year and geographic region analyzed during the period from 2011 to 2023. The populations used in the study corresponded to the intercensal and postcensal population estimates released by the IBGE for each year of the historical series [7].

### Participants

The study included individuals aged 1 year or older, of both sexes, whose records were reported as new cases of syphilis, hospitalizations due to the disease in public hospitals or hospitals affiliated with the Brazilian Unified Health System (*Sistema Único de Saúde*, SUS), or deaths for which syphilis was recorded as the underlying cause of death.

### Variables

The variables of interest were sex (male; female), age group (1-19; 20-59; ≥60 years), geographic region of residence (North; Northeast; Southeast; South; Central-West), and year of notification, hospitalization, or death (2011-2023). 

### Data sources and measurement

Anonymous, publicly available secondary data were used. Reported cases of acquired syphilis were obtained from the Notifiable Diseases Information System (*Sistema de Informação de Agravos de Notificação*, SINAN), hospitalizations due to acquired syphilis were obtained from the National Hospital Information System of the Brazilian Unified Health System (*Sistema de Informações Hospitalares do Sistema Único de Saúde*, SIH/SUS), and deaths for which acquired syphilis was recorded as the underlying cause were obtained from the Mortality Information System (*Sistema de Informações sobre Mortalidade*, SIM). Population estimates used as denominators for rate calculations were obtained from the IBGE. All data used in the study were retrieved through the TabNet tabulator available on the website of the Department of Information and Informatics of the Brazilian Unified Health System Information Technology Department (*Departamento de Informação e Informática do Sistema Único de Saúde*, DATASUS).

International Statistical Classification of Diseases and Related Health Problems 10th Revision (ICD-10) codes A51, A52, and A53 were used to identify hospitalizations and deaths due to acquired syphilis. In the SIH/SUS, hospitalizations with one of these codes recorded as the primary diagnosis were selected, whereas in the SIM, deaths with the same codes recorded as the underlying cause of death were included.

The detection rate was calculated by dividing the number of newly diagnosed cases of acquired syphilis, by location and year, by the estimated resident population of the corresponding geographic region and year of occurrence, according to IBGE data, and multiplying the result by 100,000 inhabitants. The hospitalization rate was calculated by dividing the number of hospitalizations with a diagnosis of acquired syphilis by the estimated resident population of each geographic region and year of occurrence, according to IBGE data, and multiplying the result by 100,000 inhabitants. The mortality rate was calculated by dividing the number of deaths for which acquired syphilis was recorded as the underlying cause by the resident population for the same geographic area and period, and multiplying by 100,000 inhabitants. 

### Bias

Because the study used secondary data from SINAN, SIH/SUS, and SIM, it was subject to potential biases related to underreporting, incomplete information, and inconsistencies in the records. Changes in epidemiological surveillance, expanded access to diagnosis, and variations in system coverage over the study period may also have influenced the observed trends. To minimize these limitations, official data provided by DATASUS were used, with standardized variables, indicators, and analytical procedures.

### Study size

Aggregated data for the geographic regions and for Brazil as a whole were analyzed for records of new cases, hospitalizations, and deaths due to acquired syphilis during the period from 2011 to 2023. These data were obtained from SINAN, SIH/SUS, and SIM, respectively. Detection, hospitalization, and mortality rates were calculated using annual IBGE population estimates as the denominator and expressed per 100,000 inhabitants.

### Statistical methods

The mean detection, hospitalization, and mortality rates for acquired syphilis were calculated by totaling the annual rates observed from 2011 to 2023 and dividing the result by the total number of years analyzed (n=13). Therefore, the simple arithmetic mean of the annual rates was obtained for each indicator, providing an overall measure of the average magnitude of these indicators during the study period. Due to the low frequency of deaths from acquired syphilis, mortality rates were not stratified by sex or age group for each geographic region. 

Temporal trends in detection, hospitalization, and mortality rates for acquired syphilis were analyzed using the Prais-Winsten generalized linear regression model, an appropriate method for time series data with first-order autocorrelation. The study period (2011-2023) was considered the independent variable (X), whereas the base-10 logarithm of the overall and stratified detection, hospitalization, and mortality rates for acquired syphilis was considered the dependent variable (Y). The annual percent change (APC) and its corresponding 95% confidence intervals (95%CI) were calculated for each indicator analyzed. The APC was estimated from the regression coefficient β obtained from the Prais-Winsten regression applied to the log-transformed time series (log_10_ of the rates) [8]. For this, the following formula was used: APC=(-1+10^β^)×100. This formula expresses the mean annual percentage change in each indicator over the analyzed time series. The β value represents the regression coefficient. The 95% confidence intervals for the APC were calculated in the same manner by applying the formula to the lower and upper limits of the regression coefficient (β). 

The p-value obtained for the regression coefficient (β) was used to determine the statistical significance of the observed trend, adopting a significance level of p<0.050. Trends were classified as increasing, decreasing, or stationary according to the direction and statistical significance of the regression coefficient (β). An increasing trend was defined as a positive regression coefficient (β) with p<0.050 (or a 95%CI entirely above zero); a decreasing trend as a negative regression coefficient (β) with p<0.050 (or a 95%CI entirely below zero); and a stationary trend as p≥0.050 (or a 95%CI including zero), indicating no statistically significant change over the study period.

Microsoft Excel 2019 for Windows was used for data management and for calculating detection, hospitalization, and mortality rates. All other statistical analyses were performed using Stata software, version 16.0.

## Results

From January 2011 to December 2023, a total of 1,502,384 new cases of acquired syphilis (mean detection rate: 56.4 per 100,000 inhabitants), 13,017 hospitalizations among affected individuals (mean hospitalization rate: 0.5 per 100,000 inhabitants), and 878 deaths (mean mortality rate: 0.03 per 100,000 inhabitants) due to the disease were reported in Brazil.

The detection rate in Brazil showed an increasing trend (APC 22.6; 95%CI 14.8; 30.9). The increasing trend was observed among both women (APC 22.1; 95%CI 13.7; 31.1) and men (APC 22.6; 95%CI 15.1; 30.6). This increase was consistent across all age groups evaluated, with the greatest increase observed among individuals aged 1-19 years (APC 26.6; 95%CI 14.0; 40.6) (Table 1).

The detection rate also showed an increasing trend across the Brazilian geographic regions. The largest annual increases were observed in the North region (APC 32.8; 95%CI 23.6; 42.7), followed by the South region (APC 31.8; 95%CI 14.3; 51.3). Stratified analyses by sex and age group within each region also showed increasing trends in acquired syphilis detection, particularly in the South region, where annual increases ranged from approximately 30.6% to 32.4% across all strata analyzed (Table 1).

The hospitalization rate for acquired syphilis in Brazil showed an increasing trend nationwide (APC 11.5; 95%CI 8.8; 14.3), particularly among men (APC 12.0; 95%CI 9.9; 14.2) and individuals aged 60 years or older (APC 13.9; 95%CI 11.7; 16.3) (Table 2).

An increasing trend in hospitalization rates was observed across all Brazilian regions. The largest proportional annual increase was recorded in the Central-West region (APC 28.3; 95%CI 25.6; 32.5), followed by the Northeast region (APC 12.4; 95%CI 7.7; 17.3). However, stationary trends were observed in some subpopulations. In the North region, the hospitalization rate remained stationary among individuals aged 60 years or older (APC 12.5; 95%CI −0.8; 27.6). A stationary trend was also observed among individuals aged 1-19 years in the Northeast region (APC 6.4; 95%C: −2.4; 16.1) and among individuals aged 1-19 years in the South region (APC 1.1; 95%CI −5.0; 7.5). In the remaining regions and subgroups, hospitalization rates showed increasing trends. In the Central-West region, the greatest increase was observed among women (APC 35.7; 95%CI 29.4; 42.2) and among individuals aged 20-59 years (APC 28.7; 95%CI 25.0; 32.5) (Table 2).

**Table 1:** Annual percent change (APC), 95% confidence intervals (95%CI), and temporal trend in the acquired syphilis detection rate, per 100,000 inhabitants, according to sex, age group, and region. Brazil, 2011-2023 (n=1,502,384)^a^

Variables	Total (n)	Mean rate	APC (95%CI)	p-value	Trend
Brazil	1,502,384	56.4	22.6 (14.8; 30.9)	<0.001	Increasing
Sex					
Female	587,660	43.1	22.1 (13.7; 31.1)	<0.001	Increasing
Male	909,368	69.9	22.6 (15.1; 30.6)	<0.001	Increasing
Age group (years)					
1-19	147,088	19.7	26.6 (14.0; 40.6)	<0.001	Increasing
20-59	1.224.454	79.5	22.3 (14.8; 30.2)	<0.001	Increasing
≥60	125,486	33.7	16.2 (10.0; 22.7)	<0.001	Increasing
North	94,430	40.9	32.8 (23.6; 42.7)	<0.001	Increasing
Sex					
Female	37,892	33.0	31.3 (22.6; 40.7)	<0.001	Increasing
Male	56,291	48.5	33.5 (23.5; 44.1)	<0.001	Increasing
Age group (years)					
1-19	10,627	13.3	32.1 (22.6; 42.4)	<0.001	Increasing
20-59	76,805	59.6	31.7 (22.7; 41.5)	<0.001	Increasing
≥60	6,802	31.0	34.5 (23.3; 46.7)	<0.001	Increasing
Northeast	215,944	29.9	27.9 (18.5; 38.1)	<0.001	Increasing
Sex					
Female	87,913	23.7	24.1 (14.4; 34.6)	<0.001	Increasing
Male	127,080	36.2	30.8 (20.7; 41.8)	<0.001	Increasing
Age group (years)					
1-19	20,922	9.7	27.4 (14.9; 41.2)	<0.001	Increasing
20-59	175,505	42.6	26.9 (17.9; 36.5)	<0.001	Increasing
≥60	18,843	19.6	33.2 (21.6; 45.9)	<0.001	Increasing
Southeast	754,138	67.4	18.0 (12.4; 23.9)	<0.001	Increasing
Sex					
Female	280,572	48.7	17.7 (11.8; 23.9)	<0.001	Increasing
Male	470,989	86.9	17.9 (12.4; 23.7)	<0.001	Increasing
Age group (years)					
1-19	73,851	25.4	23.9 (13.1; 35.8)	<0.001	Increasing
20-59	612,441	93.7	18.1 (12.8; 23.6)	<0.001	Increasing
≥60	65,269	38.5	9.5 (4.4; 14.9)	<0.001	Increasing
South	328,964	85.3	31.8 (14.3; 51.3)	0.001	Increasing
Sex					
Female	143,424	72.8	31.7 (14.0; 52.3)	0.002	Increasing
Male	184,656	97.7	31.0 (14.1; 50.4)	0.001	Increasing
Age group (years)					
1-19	31,365	31.5	32.4 (12.0; 56.4)	0.004	Increasing
20-59	269,247	119.8	31.2 (14.5; 50.3)	0.001	Increasing
≥60	27,468	44.6	30.6 (13.3; 50.6)	0.002	Increasing
Central-West	108,908	52.2	28.5 (19.0; 38.9)	<0.001	Increasing
Sex					
Female	37,859	35.9	26.5 (17.4; 36.4)	<0.001	Increasing
Male	70,352	68.2	29.4 (19.2; 40.5)	<0.001	Increasing
Age group					
1-19	10,353	17.2	26.9 (16.1; 38.7)	<0.001	Increasing
20-59	90,721	73.5	28.7 (18.89; 39.4)	<0.001	Increasing
≥60	7,137	28.3	23.6 (17.0; 30.5)	<0.001	Increasing

a The difference between the total number of records and the sum of the sex and age group categories arises from the existence of records with missing or unspecified information.

**Table 2:** Annual percent change (APC), 95% confidence intervals (95%CI), and temporal trend in the acquired syphilis hospitalization rate, per 100,000 inhabitants, according to sex, age group, and region. Brazil, 2011-2023 (n=13,036)^a^

Variables	Total (n)	Mean rate	APC (95%CI)	p-value	Trend
Brazil	13,017	0.5	11.5 (8.8; 14.3)	<0.001	Increasing
Sex					
Female	6,280	0.5	10.9 (7.5; 14.4)	<0.001	Increasing
Male	6,737	0.5	12.0 (9.9; 14.2)	<0.001	Increasing
Age group (years)					
1-19	1,427	0.2	8.5 (4.0; 13.2)	0.001	Increasing
20-59	9,264	0.6	11.1 (8.2; 14.1)	<0.001	Increasing
≥60	2,326	0.6	13.9 (11.7; 16.3)	<0.001	Increasing
North	534	0.2	11.0 (4.9; 17.4)	0.002	Increasing
Sex					
Female	280	0.3	9.9 (1.3; 19.3)	0.026	Increasing
Male	254	0.2	12.1 (8.1; 16.3)	<0.001	Increasing
Age group (years)					
1-19	108	0.1	10.1 (2.2; 18.6)	0.016	Increasing
20-59	367	0.3	10.6 (5.7; 15.8)	0.001	Increasing
≥60	59	0.3	12.5 (-0.8; 27.6)	0.063	Stationary
Northeast	2,477	0.4	12.4 (7.68; 17.3)	<0.001	Increasing
Sex					
Female	1,465	0.4	9.3 (3.5; 15.4)	0.004	Increasing
Male	1,012	0.3	17.6 (14.1; 21.4)	<0.001	Increasing
Age group (years)					
1-19	373	0.2	6.4 (-2.4; 16.1)	0.142	Stationary
20-59	1,740	0.5	12.2 (7.4; 17.2)	<0.001	Increasing
≥60	364	0.4	20.7 (13.0; 28.9)	<0.001	Increasing
Southeast	6,488	0.6	9.2 (6.2; 12.3)	<0.001	
Sex					
Female	2,753	0.5	8.4 (4.0; 12.9)	0.001	Increasing
Male	3,735	0.7	9.9 (7.3; 12.5)	<0.001	Increasing
Age group (years)					
1-19	571	0.2	6.9 (1.2; 13.0)	0.022	Increasing
20-59	4,659	0.7	8.6 (5.4; 12.0)	<0.001	Increasing
≥60	1,258	0.7	11.0 (8.9; 13.2)	<0.001	Increasing
South	2,169	0.6	12.3 (7.4; 17.3)	<0.001	
Sex					
Female	921	0.5	11.2 (5.2; 17.4)	0.001	Increasing
Male	1,248	0.7	12.6 (9.0; 16.3)	<0.001	Increasing
Age group (years)					
1-19	187	0.2	1.1 (-5.0; 7.5)	0.707	Stationary
20-59	1,483	0.7	11.0 (6.4; 15.7)	<0.001	Increasing
≥60	499	0.8	19.3 (12.9; 26.1)	<0.001	Increasing
Central-West	1,349	0.6	29.0 (25.6; 32.5)	<0.001	Increasing
Sex					
Female	861	0.8	35.7 (29.4; 42.2)	<0.001	Increasing
Male	488	0.5	21.9 (18.6; 25.4)	<0.001	Increasing
Age group (years)					
1-19	189	0.3	39.7 (29.6; 50.6)	<0.001	Increasing
20-59	1,015	0.8	28.7 (25.0; 32.5)	<0.001	Increasing
≥60	146	0.6	17.3 (14.5; 20.2)	<0.001	Increasing

a The difference between the total number of records and the sum of the sex and age group categories arises from the existence of records with missing or unspecified information.

Mortality due to acquired syphilis showed an increasing trend in Brazil (APC 0.8; 95%CI 0.5; 1.0), among both women (APC 0.4; 95%CI 0.3; 0.6) and men (APC 1.2; 95%CI 0.8; 1.6). An increase was observed among both adults aged 20-59 years (APC 0.7; 95%CI 0.4; 0.9) and older adults aged 60 years or older (APC 2.0; 95%CI 1.0; 3.1). In contrast, the trend among children, adolescents, and young people aged 1-19 years was stationary (APC 0.01; 95%CI −0.03; 0.05). Mortality rates increased in four geographic regions, with the greatest increase observed in the South region (APC 1.1; 95%CI 0.6; 1.7), followed by the Central-West region (APC 1.1; 95%CI 0.7; 1.6) (Table 3).

Figure 1 shows the temporal evolution of epidemiological indicators related to acquired syphilis in Brazil. An increasing trend in the detection rate of acquired syphilis was observed, with higher rates among men and individuals aged 20-59 years (Figure 1A). The hospitalization rate increased over the study period, particularly from 2016 onward, with higher rates among men and individuals aged 60 years or older (Figure 1B). Mortality due to acquired syphilis also showed an increasing trend, with higher rates among men and individuals aged 60 years or older (Figure 1C). Detection and hospitalization rates showed a declining trend in 2020.

**Table 3:** Annual percent change (APC), 95% confidence intervals (95%CI), and temporal trend in the acquired syphilis mortality rate, per 100,000 inhabitants, according to sex, age group, and region. Brazil, 2011-2023 (n=878)^a^

Variables	Total (n)	Mean rate	APC (95%CI)	p-value	Trend
Brazil	878	0.03	0.8 (0.5; 1.0)	<0.001	Increasing
Sex					
Female	276	0.02	0.4 (0.3; 0.6)	<0.001	Increasing
Male	592	0.05	1.2 (0.8; 1.6)	<0.001	Increasing
Age group (years)					
1-19	16	0.002	0.01 (-0.03; 0.05)	0.626	Stationary
20-59	418	0.03	0.7 (0.5; 1.0)	<0.001	Increasing
≥60	434	0.12	2.0 (1.0; 3.1)	0.001	Increasing
North	56	0.02	0.7 (0.5; 1.0)	<0.001	Increasing
Northeast	202	0.03	0.5 (0.3; 0.7)	<0.001	Increasing
Southeast	392	0.04	0.9 (0.5; 1.2)	<0.001	Increasing
South	154	0.04	1.2 (0.6; 1.7)	0.001	Increasing
Central-West	74	0.04	1.1 (0.8; 1.5)	<0.001	Increasing

a The difference between the total number of records and the sum of the sex and age group categories arises from the existence of records with missing or unspecified information.

**Figure 1:** Temporal trends in acquired syphilis detection, hospitalization, and mortality rates in Brazil, 2011-2023: detection rate by sex (A1), detection rate by age group (A2), hospitalization rate by sex (B1), hospitalization rate by age group (B2), mortality rate by sex (C1), and mortality rate by age group (C2). Brazil, 2011-2023

## Discussion

The analysis of epidemiological indicators for acquired syphilis in Brazil revealed increasing trends in detection, hospitalization, and mortality over the study period (2011-2023), with regional differences in the magnitude of these increases. The findings demonstrated the persistence of syphilis as a major public health problem, suggesting limitations in strategies for the prevention, diagnosis, and treatment of the infection.

The South region showed the greatest proportional increase in acquired syphilis detection in the country, in addition to increases in hospitalization and mortality rates. These findings indicated a high level of *Treponema pallidum* circulation, even in a context of broad primary health care coverage and a more structured healthcare system. The observed pattern suggested possible shortcomings in the implementation of prevention measures and the timely management of the infection, including challenges related to early diagnosis and treatment adherence, highlighting the need to strengthen local control strategies. Previous studies have already highlighted the complexity of syphilis in more developed regions, attributing the problem to the availability of healthcare services for diagnosis and treatment, persistent risk behaviors, and underreporting in some population strata [9,10]. 

The Southeast region also showed substantial increases in all three indicators, with marked rises in reported cases and in more severe outcomes, such as hospitalizations and deaths, corroborating the findings of a previous study conducted in the same region [11]. Although this region has a greater capacity for diagnosis and treatment, factors such as high population density, social inequalities, stigma, and disruptions in the supply of essential inputs have hindered infection control and contributed to increased transmission. Some studies [12,13] have shown that fragmented healthcare and socioeconomic barriers have hindered effective access to prevention and treatment even in urban centers with adequate infrastructure, thereby increasing transmission dynamics. 

In the Central-West region, the analysis revealed significant increases in acquired syphilis detection, hospitalization, and mortality. This scenario may have been associated with intense migratory processes, rapid urbanization, and insufficient expansion of healthcare services, particularly in rural areas and urban peripheries [14]. A recent study emphasized that unplanned urban growth and the deterioration of healthcare services in border areas and regions undergoing urban expansion have frequently resulted in higher rates of sexually transmitted infections, supporting the findings of the present analysis [15]. 

The North region also showed a marked increase, particularly in infection detection. The increase in reported cases may reflect both expanded access to diagnosis and a genuine increase in disease transmission. Studies have reported that structural challenges, such as limited primary health care coverage, dispersed populations, and difficulties in accessing healthcare services in remote areas and traditional communities, make the region particularly vulnerable, reinforcing the need for control strategies tailored to local realities [13,16,17]. 

The Northeast region, in turn, showed increases in detection and mortality rates but maintained a stationary trend in hospitalization rates. Although the absolute numbers in this region were lower than those observed in the other regions, the findings should be interpreted with caution, as underreporting and limited access to healthcare services may mask the true magnitude of the infection. Socioeconomic inequalities, geographic barriers, and weaknesses in epidemiological surveillance services may have led to underdiagnosis and, consequently, to the persistence of high transmission rates [18,15,19]. 

The time series showed a decline in detection and hospitalization indicators in 2020, an important finding that coincided with the first year of the COVID-19 pandemic. This decrease may have been related to the impact of the pandemic on healthcare services, including reduced testing, difficulties in accessing healthcare, interruptions in case follow-up, and possible underreporting. The subsequent increase in these indicators suggested that active transmission of syphilis persisted in the country [20]. 

The increase in national detection, hospitalization, and mortality rates underscores the importance of strengthening public policies aimed at controlling acquired syphilis. Particular emphasis should be placed on expanding access to early diagnosis, ensuring the continuous availability of medications such as benzathine penicillin, and implementing targeted educational and preventive interventions for vulnerable population groups. Furthermore, it is essential to strengthen monitoring and healthcare actions, taking into account territorial specificities and coordination among the different services within the healthcare network [21]. 

The analysis by age group and sex also revealed concerning trends. A more pronounced increase in acquired syphilis detection was observed among children, adolescents, and young people aged 1-19 years, highlighting the greater vulnerability of this population, commonly associated with early sexual initiation, inconsistent condom use, and limited access to sexual and reproductive healthcare services. Adults aged 20-59 years and older adults also showed significant increases, indicating the persistence of risk behaviors in these age groups and the lack of preventive strategies targeting aging populations [22,9].

The hospitalization findings also reinforced the severity of the situation, with significant increases observed in nearly all age groups and both sexes, suggesting shortcomings in timely diagnosis and difficulties in accessing appropriate treatment. The increase in mortality rates among adults and older adults, particularly among men, highlighted persistent inequalities in patterns of healthcare utilization, suggesting greater reluctance among men to seek medical care and adhere to treatment. These findings underscore the importance of interventions tailored to specific population groups, taking into account their epidemiological, social, and cultural characteristics to curb the progression of acquired syphilis in Brazil [23].

This study has limitations inherent to the use of secondary data from national health information systems, which are subject to underreporting, incomplete or inconsistent records, and regional differences in data quality. These limitations may have influenced the estimation of detection, hospitalization, and mortality rates for acquired syphilis, as well as the comparability of findings across the Brazilian geographic regions. The impact of the COVID-19 pandemic on healthcare services, particularly in 2020, may also have contributed to reduced testing, delayed diagnosis, and possible underreporting of cases, thereby influencing the interpretation of the observed temporal trends.

The analysis of detection, hospitalization, and mortality rates for acquired syphilis in Brazil between 2011 and 2023 demonstrated increases in epidemiological indicators across most Brazilian regions, with variations in magnitude among indicators and regions. The findings showed that syphilis remains a major public health problem, with a substantial impact on morbidity and mortality, particularly in the South, Southeast, and Central-West regions, where the highest cumulative rates and annual percentage increases were observed. The inflection in reported cases in 2020, attributed to the COVID-19 pandemic, highlighted the vulnerability of surveillance and diagnostic systems during public health emergencies, underscoring the need for resilient and sustainable strategies. The marked increase in national and regional rates pointed to persistent shortcomings in prevention, testing, and treatment efforts, as well as insufficient integration between public policies and intersectoral actions.

The results demonstrated increasing trends in acquired syphilis detection, hospitalization, and mortality in Brazil between 2011 and 2023, with important regional differences. These findings reinforce the relevance of syphilis as a public health problem and highlight the need to strengthen prevention, diagnosis, surveillance, and treatment strategies for the infection in Brazil.

## Data Availability

The data are publicly available at: https://doi.org/10.48331/SCIELODATA.CWVUG8.
